# Impact of Land-Use Change on Ecosystem Services in the Wuling Mountains from a Transport Development Perspective

**DOI:** 10.3390/ijerph20021323

**Published:** 2023-01-11

**Authors:** Yu Chen, Yilian Liu, Shengfu Yang, Chengwu Liu

**Affiliations:** 1 Department of Land Resource Management, School of Public Administration, South-Central Minzu University, Wuhan 430074, China; 2 Research Center of Hubei Ethnic Minority Areas Economic and Social Development, South-Central Minzu University, Wuhan 430074, China; 3 Department of Land Resource Management, School of Public Administration, China University of Geosciences, Wuhan 430074, China

**Keywords:** land-use change, ecosystem services, transport, Wuling Mountains, ecosystem service value

## Abstract

Transportation significantly affects regional land-use changes and ecosystem service functions. Exploring the correlations among transport development, spatial pattern of land-use changes, and ecosystem service changes are important for mitigating the deterioration of regional ecosystems due to human activities. In this study, 2000–2020 was selected as the study period to explore the effects of land-use changes on the ecosystem service value (ESV) in the Wuling Mountains. The results showed that: (1) the Wuling Mountains have experienced four stages of transport development and (2) transportation development has contributed to land-use change. The spatial pattern associated with construction land growth has evolved due to transport development. Garden land has gradually spread into the entire region with transport development. Policies from different periods have had more of an effect on ecological land and cropland. (3) During the study period, the ESV first increased and then declined. The periphery of the transportation axis formed a concentration zone of ESV cold spots. In contrast, ESV hot spots were more concentrated in areas along the Yangtze River. The results of this study provide guidance for land-use policy and spatial planning under the concept of green development.

## 1. Introduction

Transport is a precondition for human activity [1,2,3]. Human activities utilize transport as a bridge to penetrate post-developed areas, which changes land-use patterns and consequently affects the surrounding ecosystems [4,5,6]. Previous studies have explored the processes, characteristics, and drivers of land-use change (LUCC) and its effect on ecosystem services at different spatiotemporal scales using a variety of indicators and visual mapping methods [7,8,9,10,11]. However, the correlations among transport development, land-use change, and ecosystem services remain unclear. In the contemporary era of rapid transport infrastructure development, land-use policies and their spatial planning based on ecological concepts require an understanding of the effect of LUCC on ecosystem service functions from a transport development perspective.

Human activity, in the form of population, capital, technology, and information, is constantly expanding into new areas; it uses transport as a bridge and converges at nodes with favorable location conditions, leading to land-use change [12]. The results of previous studies have confirmed that transport affects LUCC. The development of roads reduces transport costs, improves accessibility, changes travel patterns, and enhances the location advantages of areas along the route. This can then increase the value of land along the route and promote the development and redevelopment of regional land, leading to an increase in the total amount of regional construction land [13]. The results of studies on the effect of expansion on ecosystem services in the middle reaches of the Yangtze River urban agglomeration have also shown that the expansion of towns along transport routes has a significant effect on ecosystem services [14]. Analyses of the spatiotemporal patterns of LUCC and driving forces in the Changzhutan urban agglomeration show that the extension of roads, railways, and other transportation networks across a region expands the scope of LUCC. Economic, policy, industrial, demographic, and urban interactions accelerate the rate of LUCC [15].

However, current research mainly focuses on the direct effects of transport on the growth of construction land [16]. There is a lack of research on the effect on other sites and the overall pattern of regional land use [17]. Based on the promotion of rural revitalization, especially in mountainous areas, the large-scale growth of garden land has become an important type of LUCC in mountainous areas and has significant effects on ecosystem services. However, in most studies, especially comprehensive LUCC studies, gardens are rarely considered as a separate LUCC type. The results of studies on agricultural ecosystems in the Three Gorges area show that mountain agriculture is transitioning from traditional food crop systems to economic crop ecosystems [18]. The results of a study on citrus plantations in Gannan revealed the formation of a 100,000 mu corridor of navel oranges based on national road No. 105, which has had a significant impact on the ecosystem service functions of the region [19]. The traditional pattern of agriculture in Von Thunen’s circles has changed due to the development of transport and multi-market supply patterns. Advances in cold chain storage technology and economic crops (gardens) have yielded agricultural zones that are no longer in close proximity to town or city centers, but clustered in medium and distant suburban areas and in zones with easy access to transport. This has significantly affected ecosystem services while changing the spatial pattern of regional land use [20,21]. Therefore, changes in traditional land-use types, i.e., cropland, construction land, forestland, grassland, and unused land, were analyzed in this study. A particular focus was placed on the large-scale growth of economic crops (gardens) in the context of rapid transport development in mountainous areas to analyze, in greater detail, the overall pattern of LUCCs in mountainous areas and their ecological effects.

Ecosystem services are goods and services that ecosystems provide for human survival, which provide a theoretical basis for understanding the interactions between human activities and ecosystems [22,23]. Costanza et al. proposed a classification system for ecosystem services and an economic valuation method [24,25,26,27]. Since then, ecosystem services have been examined worldwide. Xie et al. revised a classification system and equivalence table for ecosystem services in China based on the expert knowledge of more than 700 ecologists [28]. This led to a new approach for the valuation of ecosystem services and related research in China. Human activities are a major factor affecting the significant decline in the ecosystem service value (ESV) and ecological problems. LUCC is the most visible manifestation of human activities [29,30]. The correlation between LUCC and ecosystem services is becoming stronger; in other words, studies have demonstrated the effect of LUCC on ecosystem services [31,32]. Different land-use types, changes in land-use patterns and intensity, and different spatial scales affect ecosystem services [33,34,35,36,37,38]. Achieving the sustainable development of ecosystem services by monitoring, modeling, predicting, and decision-making with respect to LUCC has become a key concern globally. LUCC has led to the evolution of regional ecosystem services, within which transport networks can reshape the spatial distribution patterns of land use. However, in current studies, there has been less of a focus on the correlations among the transport pattern evolution, land-use spatial pattern change, and ecosystem service change. This limits our understanding of the spatiotemporal variation in the ESV based on the evolution of the overall regional spatial pattern. Therefore, a transport pattern perspective was used in this study to explore the response of spatial regional land-use patterns to transport development in different periods and the resulting changes in ecosystem services. The results of this study can provide insights into the correlations among transport, land, and ecosystem services, as well as guidance for regional land-use policy and spatial planning.

By the end of 2021, the total mileage of China’s comprehensive transportation network exceeded 6 million km, including 150,000 km of railways and 160,000 km of motorways. Based on the national “10 vertical and 10 horizontal” transport corridors, “8 vertical and 8 horizontal” high-speed railway corridors, and “71,118” national highway lines, China has formed a domestic spatial pattern of “transportation network + urban agglomeration”. This has significantly changed the spatial pattern of land use in China and significantly affected ecosystem services. However, current research focuses on relatively mature urban agglomerations in the central and eastern plains; negligible attention has been placed on the mountainous regions in the west [39,40,41,42,43]. In the past 20 years, western China has made substantial progress: the scale of highways under construction and national and provincial trunk highways has exceeded that of the eastern and central regions. There has been significant improvement in external access and depth-of-network access in western mountainous areas. Therefore, choosing the western mountainous area as the study area can better reflect the impact of the rapid development of transportation on land-use and ecosystem services in areas with complex terrain.

The purpose of this paper is to analyze three aspects: (1) the process of transport development in the Wuling Mountains since the 21st century; (2) the spatiotemporal characteristics of land-use change due to transport development; and (3) the spatiotemporal differentiation of the effect of land-use change on the ESV. This study attempts to reveal the transmission mechanism among traffic development, land-use, and ecosystem service value. We also provide countermeasures and suggestions on how to optimize land-use behavior and promote regional sustainable development under the background of rapid developments in mountain traffic. This paper is structured as follows. First, we discuss the stages of the transportation development process in the Wuling Mountains since the 21st century. Subsequently, the characteristics of LUCC in the Wuling Mountains are summarized based on spatial and nuclear density analyses. Third, the spatiotemporal variation in the effect of LUCC on the ESV is summarized based on land-use transfer matrix and hotspot analyses. Fourth, we discuss and reveal the transmission relationships among traffic, land use, and ESV, as well as the policy implications. Finally, we summarize our results and respond to the questions raised by our research.

## 2. Materials and Methods

### 2.1. Study Area

**Figure 1:**
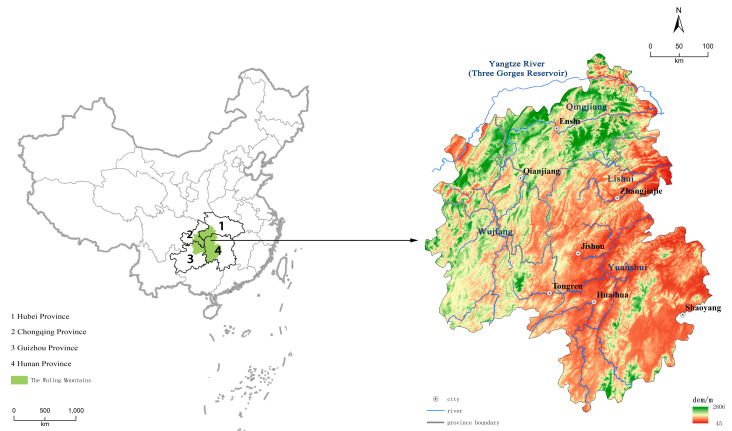
Location of the Wuling Mountains.

The Wuling Mountains are at the border of Hubei, Hunan, Guizhou, and Chongqing provinces and are part of the eastern extension of the Yunwu Mountains on the Yunnan–Guizhou Plateau. The Wuling Mountains area is the transitional zone between the two lake plains in the central and western mountainous areas. Since ancient times, it has been the bridgehead connecting Central and Southwest China (Figure 1). The Wuling Mountains have been inaccessible since ancient times. It was mainly reached by water, forming a traffic pattern with the Qingjiang, Lishui, Yuanshui, and Wujiang rivers as the main waterways. National, provincial, and county roads, as well as traditional railways, have been gradually established since China was founded. Since the 21st century, highways have gradually been constructed in the Wuling Mountains. High-speed railways have also been established in this area five years ago. The rapid development of transport has led to significant changes in land-use and ecosystem services in the region. Thus, the Wuling Mountains represent a suitable study area.

### 2.2. Data Sources

For LUCC data, Chinese land-use remote sensing monitoring data from 2000, 2005, 2010, 2015, and 2020 were used in this study, which were obtained from the Resource and Environment Science Data Centre of the Chinese Academy of Sciences (http://www.resdc.cn/DataList.aspx (accessed on 1 January 2022)). The original data were 1 km raster data. To analyze the change characteristics of land use and the ESV in the Wuling Mountains at a finer scale, a grid was used as the research unit in this study. Considering the effect of the smallest plastic cell on the research results, a 7 km × 7 km grid was selected as the basic research unit after repeatedly comparing the characteristics and differences in land-use and ecosystem service changes at different grids, followed by combining them with the visualization effect presented by the scope of the study area. The aim was to highlight the spatiotemporal variation in land-use and ESV changes at the regional scale in the Wuling Mountains. 

In reference to the land classification system of the Resource and Environment Data Centre of the Chinese Academy of Sciences and by considering the characteristics of land-use types in the Wuling Mountains, six primary land types were classified in the study area: cropland, forestland, gardens, water land, construction land, and grassland. Gardens are the result of extracting separate items of other forestlands (including all types of gardens, such as orchards, mulberry gardens, tea gardens, and medicinal materials land, among others) from the secondary classification of forestland in the land-use classification from the Chinese Academy of Sciences. Due to the large-scale cultivation of economic crops in the Wuling Mountains in the last 20 years, gardens have become an important type of LUCC in the region and have substantially affected regional ESV changes. Therefore, this category was specifically analyzed as a major land type in this study.

Basic geographic information, such as traffic data, water systems, administrative boundaries, and urban centers, were sourced from the database at the China National Mapping Center (http://ngcc.sbsm.gov.cn/ (accessed on 1 January 2021)). Economic and social-related data were obtained from local statistical yearbooks.

### 2.3. Research Methods

#### 2.3.1. Land-Use Change Measurement

Various methods can be used to measure LUCC. In this study, the LUCC and transfer matrix was utilized. The land-use transfer matrix was used to describe the transformation between land-use types at the beginning and end of the study period and could reveal the direction of the transfer between land-use types [44]. Spatial overlay analysis of the LUCC maps for the Wuling Mountains in different periods in ArcGIS was used to statistically obtain the land-use transfer matrix and determine the number of mutual land-use transformations in each period as follows:

(1) $$S_{ij}=\left[\begin{array}{c} S_{11\;}S_{12\;}S_{13\;}\cdots S_{1n}\\ S_{21\;}S_{22\;}S_{23\;}\cdots S_{2n}\\ S_{31\;}S_{32\;}S_{33}\;\cdots S_{3n}\\ \vdots \;\;\;\vdots \;\;\;\vdots \;\;\;\;\;\vdots \\ S_{n1\;}S_{n2\;}S_{n3}\;\cdots S_{n3} \end{array}\right],$$

where *S* is the area of each category, *n* is the number of land-use types, and *i* and *j* are the land-use types at the beginning and end of the study, respectively.

#### 2.3.2. ESV Calculation

Ecosystem services refer to life support products and services obtained directly or indirectly through the structure, processes, and functions of ecosystems [45]. The equivalence factor method is based on differentiating the service functions of different types of ecosystems, constructing the value equivalence of various service functions of different types of ecosystems based on quantifiable criteria, as well as assessing them in relation to the distribution area of the ecosystem. The ESV equivalent factor refers to the relative contribution of a certain service value to a certain ecosystem type (Table 1). The economic value of one ESV equivalent factor is equal to one-seventh of the national average grain yield market value in that year [46]. To obtain more realistic research results, the average grain yield (7234.5 kg/hm^2^) and average grain selling price (2.19 RMB) published by the Ministry of Agriculture and Rural Affairs of China in 2019 were used in this study. The economic value of the ESV equivalent factor in the Wuling Mountains was calculated as 2263.37 RMB/hm^2^ using the average selling price (2.19 RMB/kg) as follows:

(2) $$p=\frac{1}{7}\times a\times b,$$

where *p* is the *ESV* equivalent factor per unit area of land-use type, *a* denotes the grain production per unit area, and *b* is the average grain unit price in China in 2019. 

The equivalence factor method was first proposed by Costanza et al. and later modified by Xie Gaodi et al. as a method for valuing ecosystem services based on expert knowledge [47]. It has been widely applied in the valuation of ecosystem service functions at the sample point, regional, and national scales. This method was also selected for the assessment in this study. The value of the ecosystem services was calculated as follows:

(3) $$ESV=\sum (A_{k}\times VC_{k})\;\mathrm{and}$$

(4) $$ESV_{f}=\sum (A_{k}\times VC_{fk}),$$

where *ESV* is the ecosystem service value, *A_k_* is the area of land-use type *k*, *VC_k_* is the *ESV* coefficient, *ESV_f_* is the *f*-th ecosystem service value, and *VC_fk_* is the *f*-th service value coefficient of the *k*-th land-use type.

#### 2.3.3. Kernel Density Analysis

Kernel density analysis is a nonparametric estimation of the spatial density of elements in their surrounding neighborhoods and is used to calculate the density of point elements around each output raster image element [48]. Kernel density analysis provides a visual representation of the spatial clustering of individual LUCCs:

(5) $$f\left(x\right)=\frac{1}{nh}\sum_{i=1}^{n}K_{n}(\frac{x-x_{i}}{h}),$$

where *f*(*x*) is the kernel density calculation function at spatial location *x*, *n* is the number of points in the analysis range, *h* is the range threshold, *k* is the default weight kernel function, and *x* − *x*_i_ is the distance between points *x* and *x_i_*.

#### 2.3.4. Hot Spot Analysis

In this study, hot spot analysis (Getis–Ord Gi*) was used to detect the spatial distribution patterns of cold and hot spots formed by ESV changes in the Wuling Mountains. Based on hotspot analysis, the Getis–Ord Gi* statistics for each element in the dataset were calculated, which were used to detect where high- or low-value elements were clustered in space:

(6) $$G_{i}^{*}=\frac{\sum_{j=1}^{n}W_{ij}X_{j}-\bar{X}\sum_{i=1}^{n}W_{ij}}{\sqrt[{S}]{\frac{\left[n\sum_{j=1}^{n}W_{ij}^{2}-{\left(\sum_{j=1}^{n}W_{ij}\right)}^{2}\right]}{n=1}}},$$

(7) $$\bar{X}=\frac{\sum_{j=1}^{n}x_{j}}{n},\;\mathrm{and}$$

(8) $$S=\sqrt{\frac{\sum_{j=1}^{n}x_{j}^{2}}{n}-({\bar{x)}}^{2}},$$

where *X_i_* and *X_j_* are the values of cells *i* and *j*, respectively; *n* is the total number of samples in the study area; $\bar{X}$ is the regional mean; *W_i__j_* is the spatial weight matrix based on the neighborhood relationship of the distance function; *X* is the mean of the samples; and *S* is the variance in the samples.

If the value of the ESV change within the range is higher than that of neighboring areas, it is called a “hot spot” of ESV gain, indicating a greater increase in the value of ecosystem services within the area. If the value of the ESV change within the range is lower than that of neighboring areas, it is called a “cold spot” of ESV loss, indicating a greater decrease in the value of ecosystem services within the area.

## 3. Results

### 3.1. Development of Transport Pattern in Wuling Mountains

Considering the period of land-use data and traffic development, five years were used as the research period in this study. Transport development in the Wuling Mountains region was divided into four periods: the traditional transportation period with national roads and railways, early development period of highways with transverse lines, network period with crisscrossing highways, and period of high-speed railways.

**Figure 2:**
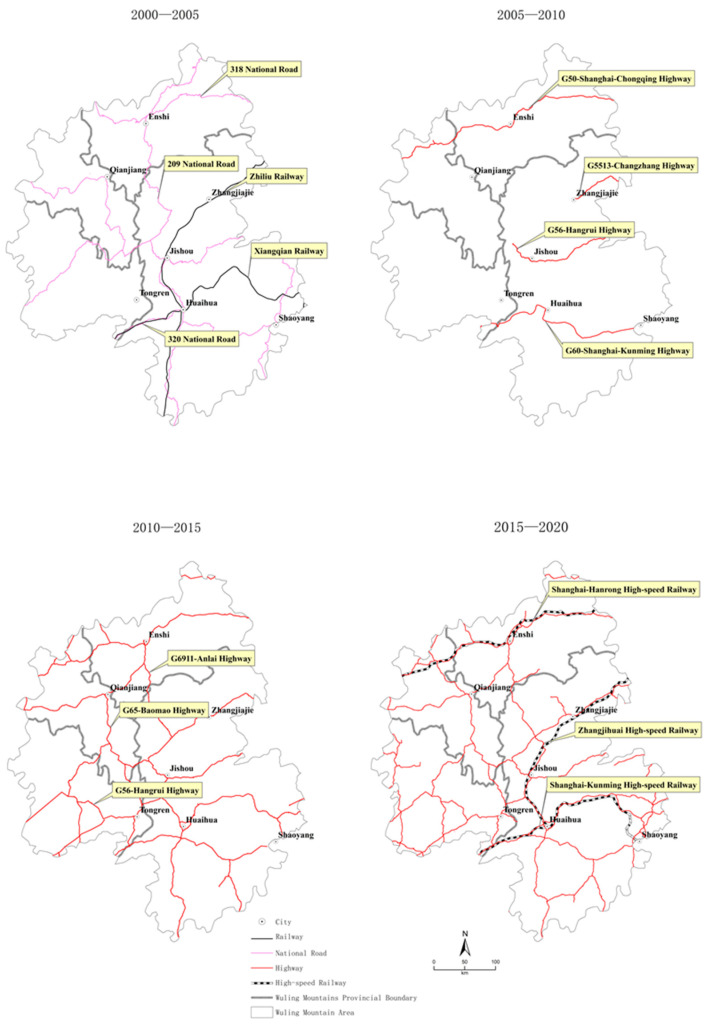
Map of the development of the transport pattern in the Wuling Mountains.

(1) Traditional transportation period (2000–2005). At the beginning of the 21st century, the Wuling Mountain area was still in the traditional traffic period. The city of Huaihua became the center of the Wuling Mountains because it was at the crossroads of two major railways and National Roads 209 and 320. Jishou and Zhangjiajie, on the Zhiliu railway, have become the capitals of their respective municipalities. Additionally, Enshi, a city in which National Road 318 and 209 meet, has become the central city in the western Hubei region. In the traditional transportation period, the Wuling Mountains formed a regional location pattern with Huaihua as the main center and Jishou, Zhangjiajie, and Enshi as regional subcenters (Figure 2).
(2) Early period of highways (2005–2010). After 2005, highways were constructed in the Wuling Mountains. From 2005 to 2010, the first highways were constructed in Huaihua (G60, Shanghai–Kunming Highway), Jishou (G56, Hang–Rui Highway), Zhangjiajie (G5513, Chang–Zhang Highway), and Enshi (G50, Hu–Yu Highway), representing the centers of the four east–west horizontal routes (Figure 2).
(3) Network period of highways (2010–2015). After 2010, the Baomao Highway G65 connected Jishou and Huaihua to the route from the eastern part of Chongqing while G56 further connected the route from the northeastern part of Qianhai to Zunyi to the west. At this point, the western part of the Wuling Mountains was connected to the national motorway network. The route from Loudi to Huaihua was added to the southeast. Vertical routes also started to develop. The north–south axis of Ensh–Lefeng–Jishou–Huaihua runs through the Wuling Mountains (G6911); highways to the northeast of Qianjiang, from Ensh to Qianjiang, and from Qianjiang to Tongren, were gradually built. By 2015, the entire Wuling Mountains region formed a motorway network with Enshi, Zhangjiajie, Jishou, and Huaihua as the central axis running north–south and connecting major node cities from east to west (Figure 2).
(4) High-speed railway period (2015–2020). After 2015, high-speed railways first connected Huaihua, Jishou, Zhangjiajie, and Enshi, whereas motorways further penetrated the remaining counties. The establishment of the Zhangjihuai, Shanghai-Kunming, and Shanghai–Hanrong high-speed railways led to a new development pattern in the Wuling Mountains region. Four major node cities were connected to the national rapid transport network through high-speed railways and their urban development further strengthened; counties also developed after being connected to the network through highways (Figure 2).

### 3.2. Land-Use Change and General ESV Changes in the Wuling Mountains

**Figure 3:**
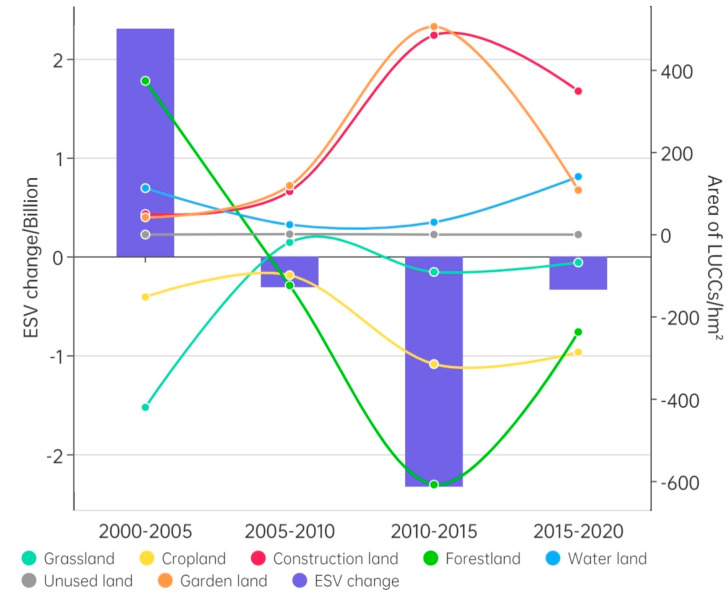
Land-use change and ESV in the Wuling Mountains from 2000–2020.

Since the 21st century, changes in the net area of construction land and gardens have been characterized by an “inverted U-shaped” curve, with both slow to high growth, followed by a slight decrease (Figure 3). The change in the net area of forestland and arable land is characterized by a “U-shaped” curve, with both peaking at a decline during 2010–2015. The difference is that forestland had a significant growth trend from 2000 to 2005. Cropland had a declining trend from 2000 to 2005. Water bodies and water facilities showed a smoother “inverted U-shaped” curve, with one end growing faster and the middle two stages growing more slowly. The net area of grassland significantly decreased in the initial stages and then continued to slightly decrease. Unused land remained relatively unchanged.

The change in the ESV during the study period was characterized by an “inverted U-shaped” curve. After a significant increase (RMB 2305 million) from 2000 to 2005, the ESV declined and peaked from 2010 to 2015 before slowing down again.

### 3.3. Analysis of Spatiotemporal Variation in Land-Use Change in the Wuling Mountains from a Traffic Pattern Perspective

#### 3.3.1. Spatiotemporal Characteristics of Land-Use Change during Different Periods of Transport Development

(1) Traditional transportation period (2000–2005)

**Figure 4:**
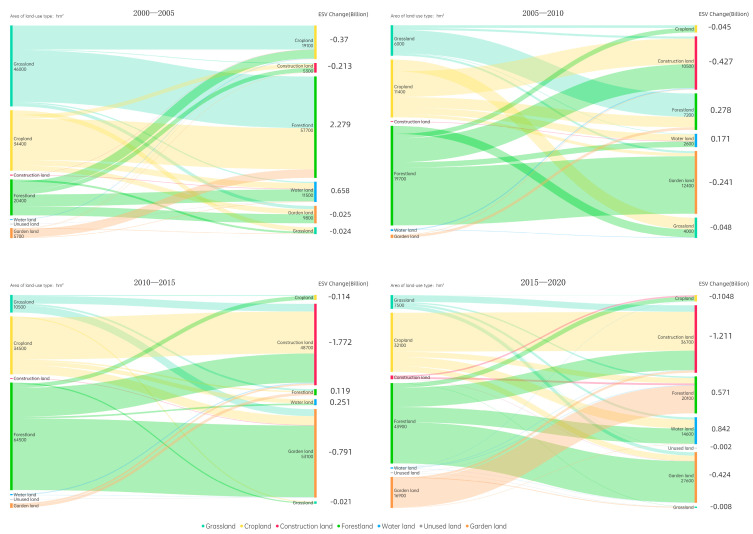
Mulberry map of land-use changes in the Wuling Mountains by period.

After the megaflood in the Yangtze River Basin in 1998, ecological restoration projects began in the middle and upper reaches of the Yangtze River Basin, with a large number of mountainous areas being returned to forests from farmland and grassland while plains areas were returned to lakes from fields. Therefore, the most important feature of LUCC from 2000 to 2005 in the Wuling Mountains was the conversion of arable land and grassland to forestland, mainly in Chongqing and Guizhou along the Yangtze River. Additionally, the construction of water conservancy facilities, as represented by the Three Gorges Water Conservancy Project, led to an increase of 11,500 hectares (Figure 4). Traditional national roads and railways had less of an impact on construction land during this period; construction land grew slowly around key cities and transportation hubs. The number of gardens started to grow in the Huaihua, Shaoyang–Loudi, and Enshi areas.

(2) Early period of highways (2005–2010)

**Figure 5:**
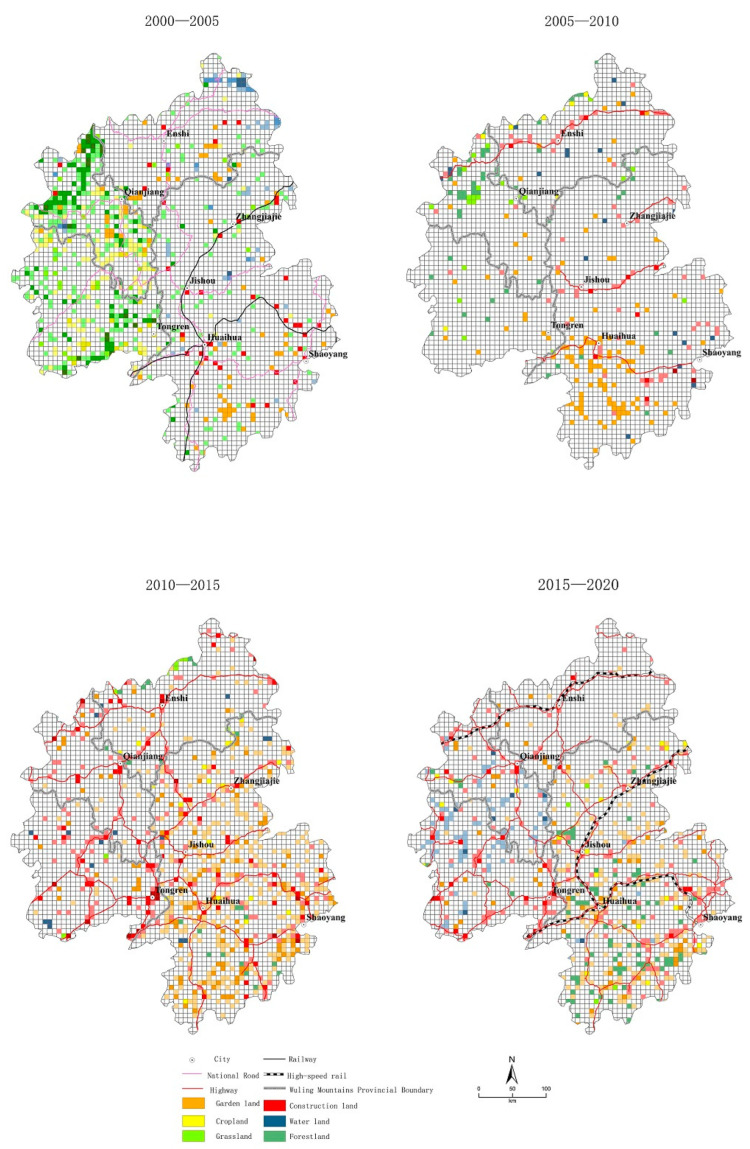
Spatial distribution of land-use changes from 1980–2020.

After 2005, the Wuling Mountains region entered an era of highway construction. The construction of four horizontal highways, i.e., the Chang–zhang Highway (G5513), Shanghai–Kunming Highway (G60), HangzhouRuili Highway (G56), and Shanghai–Chongqing Highway (G50), led to the rapid growth of construction land along the route. Additionally, the enhancement of transportation reduced transportation costs and expanded the sales market. Therefore, transportation has had a positive effect on the growth of gardens. During this period, garden land considerably increased by 12,400 ha and was mainly located in the Huaihua and Shaoyang regions, with relatively favorable transport conditions. Generally, a large amount of forestland was transformed into gardens and construction land; a large amount of cropland was transformed into construction land and grassland, both of which generally decreased (Figure 4). A portion of new forest from cropland is still being maintained to the east of Chongqing along the Yangtze River (Figure 5).

(3) Network period of highways (2010–2015)

After 2010, highways extended into the western part of the Wuling Mountains, with the formation of a network pattern, including both vertical and horizontal routes. Construction land in the Wuling Mountains also showed a network growth pattern along the highway perimeter during this period, with a considerable increase of 48,700 ha. The overall development of transport also led to a considerable increase of 53,100 ha in gardens (Figure 4). Based on the network of highways across the region, garden growth was distributed along the transportation perimeter across the region (Figure 5). In contrast, cropland and forestland markedly decreased during this period due to the massive conversion to construction land and gardens.

(4) High-speed railway period (2015–2020)

The Wuling Mountains region entered the high-speed railway era in 2015 with the establishment of three high-speed railways, further solidifying the hub positions of Enshi, Zhangjiajie, Jishou, and Huaihua in the Wuling Mountains region. After a period of rapid growth in construction land, this growth slowed down, but remained at a high level, with a total increase of 36,700 ha (Figure 4). The spatial distribution was “polarized”, with growth concentrated around major transport hubs, particularly Huaihua, Tongren, Jishou, and Shaoyang, with high-speed railways passing through these regions (Figure 5). Similar to the growth of construction land, the growth of garden land decreased to 27,600 ha, which was mainly distributed around transport hub cities. Although the total amount of forest and arable land decreased, the reduction declined and some growth in forestland and cropland still occurred in the Yuan River Basin. The watershed and water conservancy facilities in the Wujiang River Basin have increased substantially.

#### 3.3.2. Analysis of Spatiotemporal Variation in Kernel Density of Each Land-Use Change

**Figure 6:**
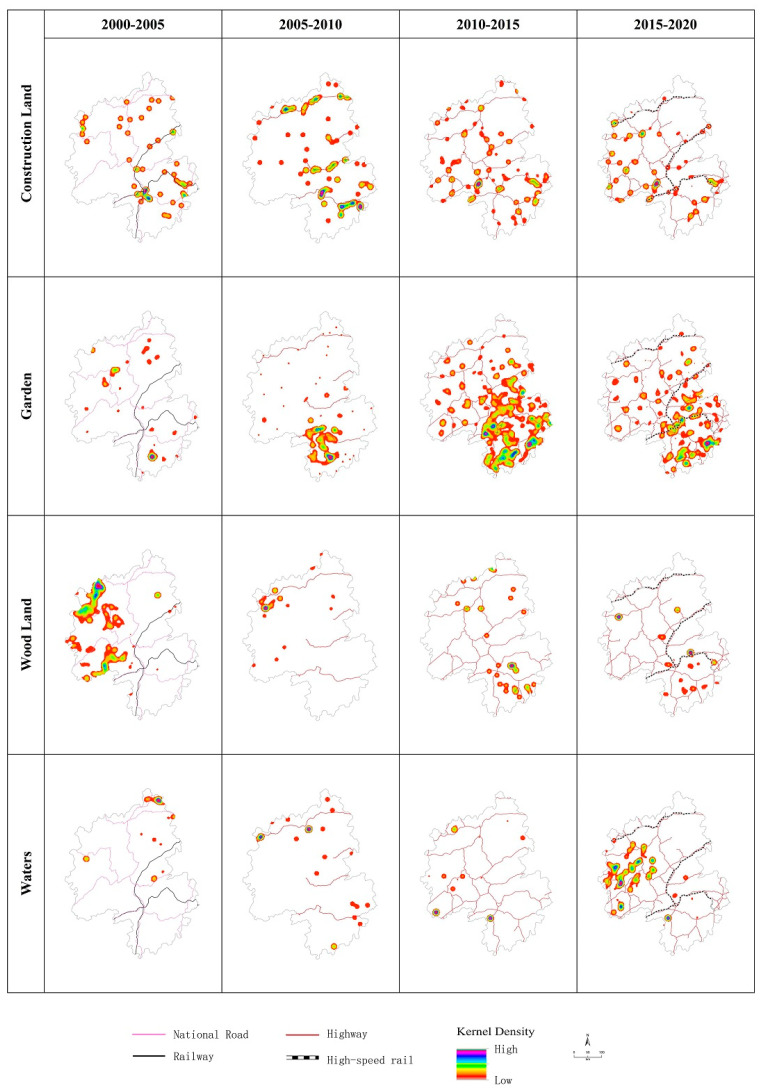
Kernel density analysis of the spatial changes in major land types.

The kernel density analysis method allows for a summary of the patterns of spatiotemporal changes in the growth of various types of sites. The spatial distribution of high-value kernel density areas with increased construction land significantly correlates with the development of traffic axes. The overall pattern evolves with the development of traffic routes in a “point–line–network–pole” process (Figure 6).

The spatial distribution of high-value areas with increased kernel density in garden land is also affected by the development of transport. Garden land, as an economic crop, must be developed on a scale that reduces transport costs. It has also expanded with the development of transportation (Figure 6). However, garden land is constrained by natural geographical conditions. The spatial distribution pattern does not indicate a significant correlation with the traffic axis, as observed for construction land. It starts from a plain area with good traffic (Huaihua and Shaoyang; gradually extending to the entire area).

Changes in woodland, as the main type of ecological land, are strongly affected by macro policies, especially after the 1998 megaflood in the Yangtze River Basin. Large-scale ecological restoration projects have been carried out along the Yangtze River Basin to return farmland to forests and lakes. Thus, the high-value area of the kernel density for the increase in woodlands was initially concentrated along the Yangtze River in the western part of the Wuling Mountains and gradually narrowed and advanced to the southeast with policy implementation (Figure 6).

Land for water and water facilities is related to the advancement of water conservancy projects in the Wuling Mountains. Since the 21st century, a large number of water conservancy projects, such as the Three Gorges Project, have been implemented in the mountains. Overall, the increase in the kernel density in water areas is a gradual process, which has been expanding from individual points in the Three Gorges Reservoir area to other areas (Figure 6).

### 3.4. Effect of Land-Use Change on Changes in ESV in Wuling Mountains

#### 3.4.1. Spatiotemporal Variation in ESV in Different Periods of Transport Development

(1) Traditional transportation period (2000–2005)

**Figure 7:**
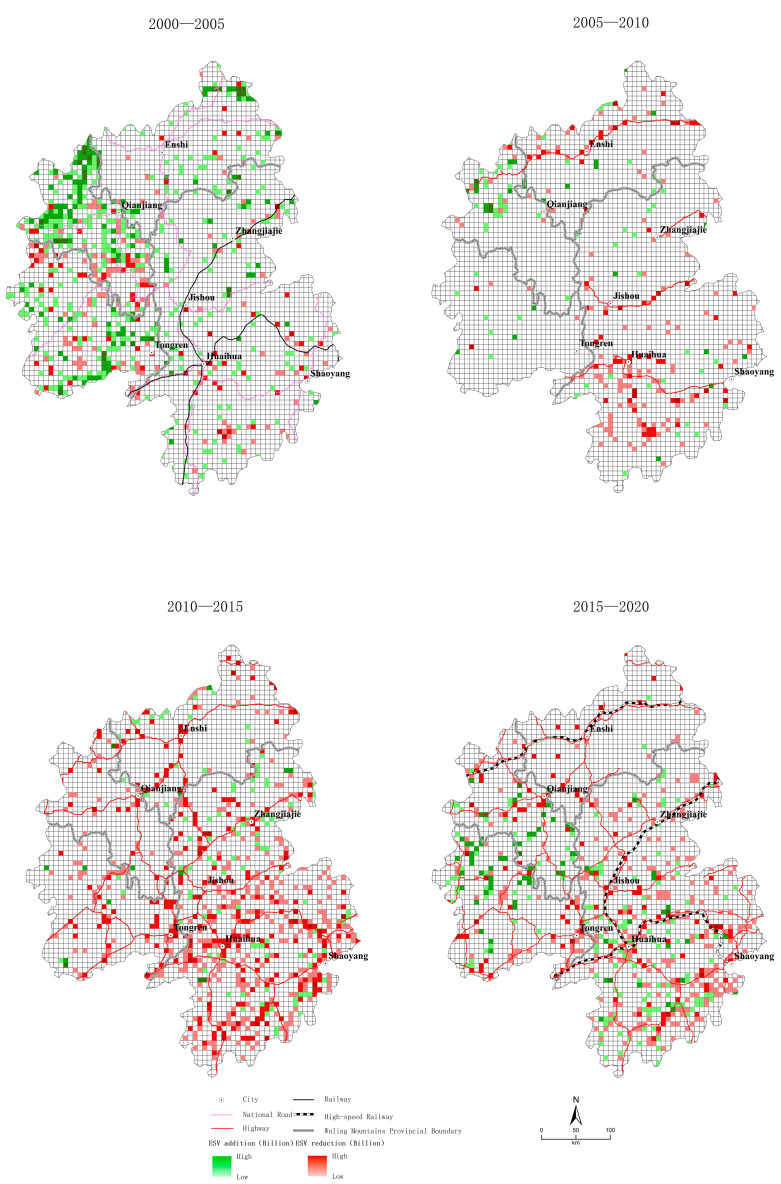
Spatial differentiation of the ecosystem service values in the Wuling Mountains.

From 2000–2005, the total ESV growth of RMB 2304 million mainly originated from the growth of forestland and water, contributing RMB 2278 billion and RMB 657 million (Figure 4), respectively, with water concentrated in the Three Gorges Reservoir area of the Yangtze River and forestland concentrated in the Yangtze River region along the eastern part of Chongqing and northeastern Guizhou (Figure 7). The increase in cropland remained the main source of ESV reduction, contributing RMB 371 million, which was concentrated in northeast Guizhou. The increase in construction land had a relatively weak effect on ESV reduction, contributing RMB 212 million, which was concentrated in the Huaihua and Shaoyang regions.

(2) Early highway period (2005–2010a)

From 2005 to 2010a, the overall decrease in the ESV was RMB 310 million, which was mainly due to the increase in construction land (accounting for RMB 425 million). Construction land was mainly located around the four highways that were first built in the Wuling Mountains. The second largest source of the decrease in the ESV was the increase in garden land, accounting for RMB 241 million (Figure 4). It was mainly distributed in the Huaihua area, which had relatively superior traffic conditions during the early period. The main source of the increase in the ESV was still the increase in forestland, which contributed RMB 279 million and was concentrated in the area along the Yangtze River in the eastern part of Chongqin (Figure 7).

(3) Network period of highways (2010–2015)

From 2010 to 2015, the overall significant decrease in the ESV of RMB 2327 million was mainly derived from the large increase in construction land (accounting for RMB—1771 million) (Figure 4), which was distributed around roads across the region with networked highways. Another part of the decrease originated from the massive increase in garden land (accounting for RMB—791 million). Garden land was also distributed across the entire region due to the improvement in traffic conditions. The increase in the ESV was mainly due to water bodies and water facilities (accounting for 251 million), mainly in northeastern Guizhou (Figure 7).

(4) High-speed railway period (2015–2020)

During this period, the overall regional ESV decreased by RMB 337 million. It mainly originated from the growth of construction land (accounting for RMB—1.21 billion) and garden land (accounting for RMB—425 million) (Figure 4). The growth of construction and garden land was the main reason for the reduction in the ESV. They were located along and around the entire area of traffic and in the Huaihua area with relatively superior traffic conditions (Figure 7). The growth of forestland and water areas constituted the main source of ESV growth during this period. Forest land was mainly converted into cultivated and construction land. Water areas were mainly converted from arable land to forestland. The two highest ESV types contributed to an ESV growth of RMB 570 million and RMB 842 million, respectively. Therefore, compared with the dramatic reduction of the ESV in the previous period, the positive effect of the growth of forestland and water areas slowed the overall regional ESV reduction.

#### 3.4.2. Hot-Spot Analysis of ESV Changes

The results of the hot-spot analysis showed that the ESV had a spatial agglomeration effect in all periods. Based on the overlay analysis of the traffic development process, we determined the following characteristics.

**Figure 8:**
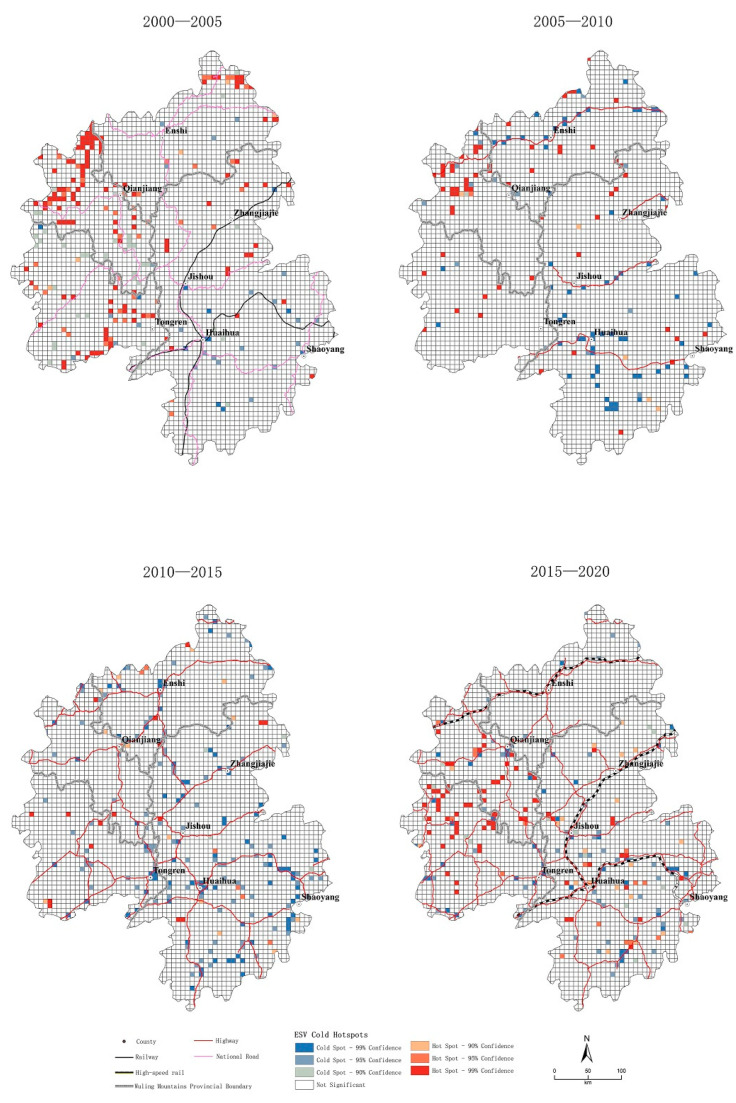
Hot-spot analysis of ESV changes.

(1) The cold-spot area, in which the ESV reduction value was always concentrated, showed a notable correlation with traffic; it was distributed in the vicinity of the main traffic arteries in each period. When traffic was used as the axis, a “point–line–network–pole” trend could be observed. From 2000 to 2005, cold spots were scattered around the hub cities of traditional railway lines (e.g., Huaihua, Loudi, Jishou, and Cili) in the form of dots. From 2005 to 2010, with the construction of the four highways, the cold-spot area was primarily distributed around the highways. Overall, the cold-spot cluster area presented four horizontal linear spatial patterns. From 2010 to 2015, the highways were further connected to form a network; the cold spot cluster area showed a spatial network pattern. From 2015 to 2020, the decreased growth of construction land led to a decrease in cold spots compared with the previous period. However, owing to the establishment of the high-speed railway, the surrounding hub cities along the line (e.g., Huaihua, Jishou, Shaoyang, and Tongren) were still the main areas of cold-spot agglomeration. The overall form of the cold-spot agglomeration area showed a polarized spatial pattern (Figure 8).
(2) Hot-spot areas of ESV value-added clustering were more related to geographical location factors. The areas surrounding the Yangtze River have always been the key areas for hot-spot clustering. The Wuling Mountains are located in the middle and upper reaches of the Yangtze River; Hubei, Chongqing, and Guizhou are adjacent to the Yangtze River. After the 1998 Yangtze River flood, large-scale ecological restoration projects were carried out along the Yangtze River Basin to return farmland to forests and lakes. Additionally, the Three Gorges Project represents the rapid development of water conservancy projects in the past 20 years. With these two policy backgrounds, the Yangtze River has become an ESV hot spot. According to the implementation of policy phases, the temporal sequence of the hot-spot area range showed a “large-scale expansion–small-scale concentration–shrinkage–repeated expansion” pattern (Figure 8).

## 4. Discussion and Insights

### 4.1. Effect of Land-Use Change on ESV from a Transport Pattern Perspective

Heri Lefebvre states that “(the production of space) begins with the use of existing space, such as waterways; then comes roads; then follows the construction of railways; and most recently, highways and airports [49]”. Capital, population, and land, which are the basic elements of productivity development, move into new areas with transportation. Regions along transport routes, particularly hub towns, can more efficiently accumulate these elements [50]. Many studies have shown that LUCCs along transport routes are more dramatic and have the most direct effect on construction land [51,52,53]. The spatiotemporal differentiation in the LUCC and kernel density analysis in this study showed that as transportation continued to improve, a “point–line–network–pole” growth trend developed with the hub city and transportation as the axis of construction land.

Additionally, the effect of transport on agricultural land indirectly affects economic cropland (gardens) within agricultural land. The von Thunen agricultural location theory demonstrates the correlation between economic crops and town distance, i.e., the role of transportation costs in the spatial distribution of economic crops [54]. In modern society, with the development of cold chain preservation technology and diversification of the consumer market, the von Thunen circle of the agricultural production space also presents various changes [55]. This is illustrated in this study via the kernel density analysis: gardens grew slowly and were scattered during the traditional transport period, mainly concentrated in the Huaihua–Shaoyang area with railways and national roads. Rapid expansion began in the period of highways, which was initially concentrated in the Huaihua–Shaoyang area, in which highways were first constructed. With growth in the high-speed network, gardens expanded to the northern and western Wuling Mountains and eventually to the entire area.

The Wuling Mountains are a mountainous region with forestland as the main land-use type. The growth of construction land and gardens mainly originated from forestland, which was the main reason for the ESV reduction. Therefore, in terms of spatial distribution, the area around the traffic axis was the main area for ESV reduction. Spatial clustering analysis showed that the spatial distribution of cold spots exhibited a “point–line–network–pole” trend.

Forestland, water areas, and cultivated land were more affected by macro-policies because they are directly related to ecology and food security. Since the 21st century, China has promoted reforestation and water conservation projects. The Wuling Mountains are located in the middle and upper reaches of the Yangtze River region. After the great flood of 1998, reforestation and water conservancy construction phases continued to advance [56]. The hot-spot analysis showed that the spatial evolution of the ESV was characterized by a phased “large-scale expansion–small-scale concentration–shrinkage–repeated expansion” pattern in the hot-spot area of the western Wuling Mountains region along the Yangtze River.

In summary, the impact of transportation on land-use changes and ecosystem services is essentially an important node where all productivity factors converge to the traffic axis, where transportation infrastructure is the conduction path. The surrounding areas of these nodes became the focal point for labor, capital, technology, and other factors. The agglomeration of these productivity factors is manifested in the spatial transformation from land-use types with relatively low economic value (forest land, cultivated land, and grassland, among others) to land-use types with higher economic value (construction land and garden land). However, the ESV of land-use types with a high economic value is usually relatively low, which further leads to a change in the ESV from high to low in these regions. This is the transmission mechanism for the impact of transport development on land-use and ecosystem services. Therefore, at a macro level, the evolution of the spatial pattern of the ESV is manifested as hot spots that constantly reduce spread with the traffic axis, whereas hot spots that increase in the periphery expand or shrink in stages due to policy changes.

### 4.2. Policy Applications

#### 4.2.1. Transportation and Land Use Optimization

This study shows that transportation plays a notable role in land-use change in the node area along the route. The Wuling Mountains are a transition zone from central China to the southwest. The topography of the region connects the first step (the Two Lakes Plain) and the second step (the Yungui Plateau). After the 1950s, there was increased road development. At this time, cities along these roads further developed while some cities that had historically developed due to water transportation fell into a period of decline. One of the most typical examples is Huaihua. Huaihua has remained a small city in history owing to its distance from major water transport lines. With a successively increasing number of roads and railways passing through Huaihua, it quickly became an important node city in the region; its administrative level was also upgraded to the highest level in the region [57]. The development of other adjacent cities, which once thrived on water, has declined. With the substantial development of China’s infrastructure after the 21st century, the construction of highways and high-speed railways gradually began in the Wuling Mountain area, during which the city (construction land) and its surrounding cash crops (garden land) entered a stage of rapid expansion. However, at the same time, this also led to a decrease in the ESV in these areas. Therefore, from an urban development perspective, we should seize the opportunities resulting from traffic development. However, from a security perspective in the overall regional ecosystem, the scale of the city should be properly controlled, especially in mountainous areas. As the periphery of the city is mainly dominated by woodland and other land with high ESVs, we must prevent the disorderly expansion of the city.

#### 4.2.2. Sustainable Development

This study shows that areas surrounding transportation lines are agglomeration areas with a decrease in the ESV. In contrast, ESV growth in the vast surrounding areas depends on the guidance of relevant policies. Therefore, managing the relationship between economic development and ecological protection is the key to this problem. First, sustained investment in environmental protection and restoration projects is necessary. In mountainous areas, returning farmland to forests, natural forest protection projects, and water conservancy projects based on clean energy are important sources of improving the regional ESV. Second, we should establish a sustainable cash flow for ecological protection projects. Ecological protection projects require a large amount of capital input, which itself cannot produce direct economic benefits. In the past, China’s ecological conservation projects mainly relied on financial input; however, these projects now require the establishment of market mechanisms [58]. This study confirmed that economically beneficial areas tend to have a negative impact on ecosystem services while less economically developed areas tend to bear the burden of ecological conservation. From an entire regional perspective, the economically developed areas also enjoy the spillover effect of ecological protection from economically backward areas. Therefore, we must establish a set of ecological compensation mechanisms based on the regional scale. Economic benefit areas should provide funds to the supply regions associated with ecosystem services through reasonable market mechanisms. This can establish the sustainable cash flow required by the construction of ecological protection projects.

### 4.3. Limitations

In this study, the ecological effects of LUCC in the Wuling Mountains were explored at a macroscopic scale using a 7 km × 7 km grid as the study scale. This scale is suitable for large areas in the Wuling Mountains. However, we note that the ecological effects of land use are scale dependent, especially at the microscale, i.e., the mechanisms behind the ecological effects of LUCC at a finer scale may be different. As the original land-use data in this paper is 1 km, accurately explaining the mechanism at the micro scale is difficult. Future studies should use a finer scale. Additionally, spatial clustering statistical analysis methods, such as kernel density and hot-spot analysis, were used in this study to explore the spatiotemporal differentiation of land use and ESVs. The correlation between them was analyzed using spatial superposition analysis. However, based on the results of this study, the impact of traffic on land-use and ecosystem services is limited by distance: this impact is notably greater in areas around the traffic axis. Therefore, buffer zone analysis or spatial regression analysis should be used to examine the distance of the impact of traffic on land-use and ecosystem services.

## 5. Conclusions

This study is based on land-use and transport data from 2000, 2005, 2010, 2015, and 2020a. The spatiotemporal divergence patterns of LUCC and ESV and the correlation between them were explored from a transport development perspective in the Wuling Mountains using the land-use transfer matrix, kernel density, and hot spot analyses. Based on the results, we obtained the following conclusions.

(1) Since the 21st century, in five-year increments, the development of transportation in the Wuling Mountain areas has roughly experienced four stages: the traditional transportation period, early expressway development, expressway networking period, and high-speed railway period.
(2) The development of construction land and gardens first declined rapidly, then increased, and finally declined slightly. Forestland and water bodies have experienced rapid development, weakening, slowing, and finally more rapid development. In terms of spatially divergent characteristics, the development of transport has had a significant effect on construction land and indirectly affects land for economic crops (garden). The spatial pattern of construction land growth has evolved along with transport development in a “point–line–network–pole” process. Gardens have also gradually spread from the better-connected southeastern region to the entire region. In contrast, forestland has been influenced by the policies of returning farmland to forestry and ecological civilization. It has significantly expanded from the Yangtze River coastal area, then shrank, and then gradually spread across the entire region.
(3) In response, the ESV increased, decreased, significantly declined, and then the decline slowed over the past 20 years. In terms of spatial pattern changes, the periphery of the transportation axis has formed a cluster of ESV cold spots. The spatial pattern also showed “point–line–network–pole” evolution characteristics due to the development of transportation. In contrast, ESV hot spots were more concentrated in areas along the Yangtze River. The regional scope showed “large scale expansion–small scale concentration–shrinkage–repeated expansion”.

This study revealed the transmission mechanism among transportation, land use, and the ESV. The factors of productivity converged to the important nodes of the traffic axis with the transport infrastructure as the conduction path, thus triggering the growth of land with high economic benefits, such as construction land and garden land. This also led to a reduction in the ESV in the area around the traffic axis. The periphery of the traffic axis was mainly affected by ecological policies, which determine the growth of ecological land use and the ESV. Therefore, we must control the scale of urban development while seizing opportunities associated with traffic development. A sustainable cash flow between the ESV supply and demand regions can be formed through market mechanisms, such as regional ecological compensation, to support the investment demand of regional ecological protection projects.

## Figures and Tables

**Table 1:** Ecosystem services value coefficients in the Wuling Mountains (yuan/hm^2^).

The First Category	The Second Category	Grassland	Cropland	Construction Land	Forestland	Water Land	Unused Land	Garden
Provisioning service	Food production	973.3	2263.4	0.0	746.9	1199.6	45.3	1516.5
	Raw material produce	814.8	882.7	0.0	6744.8	792.2	90.5	3825.1
Regulating service	Gas regulation	3395.1	1629.6	0.0	9777.8	1154.3	135.8	5703.7
	Climate regulation	3530.9	2195.5	0.0	9211.9	4662.5	294.2	5703.7
	Hydrological regulation	3440.3	1742.8	0.0	9257.2	42,483.5	158.4	5500.0
	Waste disposal	2987.7	3146.1	0.0	3893.0	33,611.0	588.5	3530.9
Supporting service	Soil conservation	5070.0	3327.2	0.0	9098.8	928.0	384.8	6224.3
	Biodiversity	4232.5	2308.6	0.0	10,207.8	7763.4	905.4	6269.5
Cultural service	Providing aesthetic Landscape	1969.1	384.8	0.0	4707.8	10,049.4	543.2	2557.6
A total of (Yuan/hm^2^)		26,413.5	17,880.6	0.0	63,646.0	102,643.8	3146.1	40,763.3

## Data Availability

The data presented in this study are available on request from the corresponding author.
